# Corrigendum to “Estimation of maximal oxygen consumption and heart rate recovery using the Tecumseh sub-maximal step test and their relationship to cardiovascular risk factors” [Artery Research 18 (June 2017) 29–35]

**DOI:** 10.1016/j.artres.2018.07.003

**Published:** 2018-09

**Authors:** Alun D. Hughes, Nish Chaturvedi

**Affiliations:** Institute of Cardiovascular Sciences, University College London, London, WC1E 6BT, UK

The authors regret that the published article contained an error in Eqs. (2) and (3). The correct equations should be:

(2) $$\left(Men\right)\;VO_{2}\max\left(AVD\right)=1.29\cdot \sqrt{\frac{Load\left[kg.m.\mathrm{mi}n^{-1}\right]}{Exercise\;heart\;rate\left[bpm\right]-60}}\cdot e^{-0.0088\cdot age\left[y\right]}$$

(3) $$\left(Women\right)\;VO_{2}\max\left(AVD\right)=1.18\cdot \sqrt{\frac{Load\left[kg.m.\mathrm{mi}n^{-1}\right]}{Exercise\;heart\;rate\left[bpm\right]-60}}\cdot e^{-0.0090\cdot age\left[y\right]}$$

The authors would like to apologise for any inconvenience caused.

